# Crystal structure of (K_1.5_Na_0.5_)Ca_3_Si_3_O_10_

**DOI:** 10.1107/S2056989022006193

**Published:** 2022-06-16

**Authors:** Volker Kahlenberg

**Affiliations:** a University of Innsbruck, Institute of Mineralogy & Petrography, Innrain 52, A-6020 Innsbruck, Austria

**Keywords:** crystal structure, cation substitution, solid solution, potassium sodium calcium silicate

## Abstract

Single crystals of (K_1.52_Na_0.48_)Ca_3_Si_3_O_10_ [idealized (K_1.5_Na_0.5_)Ca_3_Si_3_O_10_, di(pot­assium, sodium) tricalcium tris­ilicate], were obtained from the crystallization of a glass with a molar oxide ratio of K_2_O:Na_2_O:CaO:SiO_2_ = 1.5:0.5:6:6 that was annealed at 1273 K. The crystal structure can be characterized as a mixed-anion oxidosilicate with isolated [SiO_4_] tetra­hedra as well as [Si_4_O_12_] *vierer* single rings. The insular and cyclic silicate anions occur in the ratio 2:1, resulting in the idealized crystallochemical formula K_3_NaCa_6_[SiO_4_]_2_[Si_4_O_12_]. Charge compensation is provided by K^+^, Na^+^ (occupying the same sites) and Ca^2+^ cations. The two unique Ca^2+^ cations are coordinated by six O atoms, forming distorted octa­hedra. By sharing common corners, edges and faces, these [CaO_6_] polyhedra build up octa­hedral layer-like motifs parallel to (010). (K/Na)^+^ ions reside in voids between the the silicate anions and the calcium centered octa­hedra and are coordinated by eight to nine oxygen ligands. (K_1.5_Na_0.5_)Ca_3_Si_3_O_10_ is isotypic with K_2_Ca_3_Si_3_O_10_ indicating the existence of a solid-solution series K_2–*x*_Na_*x*_Ca_3_Si_3_O_10._

## Abstract 2

A new phase in the quaternary system K_2_O–Na_2_O–CaO–SiO_2_ with idealized composition (K_1.5_Na_0.5_)Ca_3_Si_3_O_10_ has been structurally characterized. The results indicate the existence of a solid-solution series K_2–*x*_Na_*x*_Ca_3_Si_3_O_10_.

## 1. Chemical context

The system K_2_O–Na_2_O–CaO–SiO_2_ has attracted considerable inter­est in materials science due to its relevance to glass technology. Actually, the majority of mass-produced flat and hollow glasses that are ubiquitous in our everyday life are based on melts where the above-mentioned four oxides are the main components (Varshneya, 1994 ▸; Shelby, 2009 ▸). On the other hand, there are also some archaeologically relevant glass types where this system comes into play. Examples include ancient Chinese glassware that can be dated back to the so-called Warring States Period, 475–220 BC (Liu *et al.*, 2015 ▸), as well as wood ash or forest glasses that were produced in Europe during the Middle Ages (Wedepohl & Simon, 2010 ▸). A more recent field of inter­est where the system K_2_O–Na_2_O–CaO–SiO_2_ is of importance are silicate-oxide slags and ashes occurring during the combustion of biomass fuels. The composition of the silicate fraction of these residuals can be represented in the *pseudo*-ternary diagram K_2_O(+Na_2_O)–CaO–SiO_2_. Consequently, the corresponding simplified ternary phase diagram K_2_O–CaO–SiO_2_ has been frequently used for the inter­pretation of (i) melt formation/composition and (ii) the phase content at sub-solidus conditions (*e.g.* Lindberg *et al.*, 2013 ▸). It is well known that a large number of synthetic and also some natural *ternary* sodium/potassium calcium silicates exist (Schmidmair *et al.*, 2015 ▸; Kahlenberg & Hösch, 2002 ▸, and references cited therein). For the *quaternary* compounds, which may also occur in the ash deposits, the situation is less well understood. So far, the existence of only three anhydrous compounds has been proven (Kahlenberg *et al.*, 2018*a* ▸,*b* ▸). Consequently, there is a clear need for thorough experimental determinations of the phase relationships, starting with a comprehensive survey of the existing compounds and the determination of their crystal structures in order to decipher what happens in the sub-solidus region. The present article is a further step into this direction.

## 2. Structural commentary

**Figure 1:**
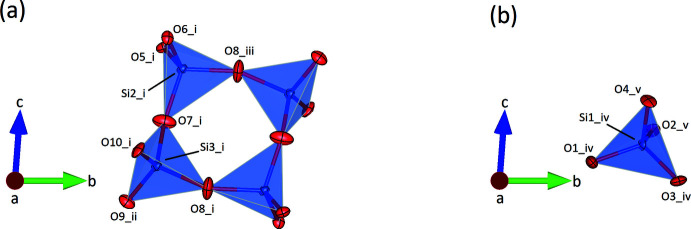
(*a*) [Si_4_O_12_] *vierer* single ring and (*b*) insular [SiO_4_] tetra­hedron in the crystal structure of (K_1.5_Na_0.5_)Ca_3_Si_3_O_10_. The projection is parallel to [100]. Oxygen and silicon atoms are shown in red and blue, respectively. Displacement ellipsoids are drawn at the 70% probability level. [Symmetry codes: (i) *x*, *y* − 1, *z*; (ii) 1 − *x*, −*y*, −*z*; (iii) 1 − *x*, 1 − *y*, −*z*; (iv) 1 + *x*, *y*, *z*; (v) 2 − *x*, 1 − *y*, 1 − *z*]

(K_1.5_Na_0.5_)Ca_3_Si_3_O_10_ belongs to the group of so-called mixed-anion silicates. The triclinic compound contains insular [SiO_4_] as well as cyclic [Si_4_O_12_] units in the ratio 2:1. Actually, three crystallographically independent silicon atoms can be distinguished: Si1 represents the center of the insular (*Q* ^0^-type) tetra­hedron while Si2 and Si3 are linked into a *Q* ^2^-type *vierer* single ring, whose barycenter is located on a center of inversion (Fig. 1 ▸ *a*,*b*). Consequently, the four silicon atoms of a single ring reside in the same plane. The Si—Si—Si angles within the ring are almost 90° [Si2—Si3—Si2(1 − *x*, 2 − *y*, −*z*) = 88.87 (6)° and Si3—Si2—Si3(1 − *x*, 2 − *y*, −*z*) = 91.14 (6)°]. The four tetra­hedra exhibit an *UUDD* sequence of up (*U*) and down (*D*) pointing vertices. Si—O bond lengths and O—Si—O bond angles are in the normal range observed in oxidosilicates (Liebau, 1985 ▸). The two independent Si—O—Si angles of the rings have values of 148.7 (4) and 156.4 (3)°, respectively. Both are significantly larger than the value of 140°, which is assumed to correspond to an unstrained Si—O—Si angle (Liebau, 1985 ▸), indicating that the cyclic anion is under tension. The geometric distortions of the tetra­hedra can be expressed numerically by means of the quadratic elongation *λ* and the angle variance *σ* ^2^ (Robinson *et al.*, 1971 ▸). These parameters have the following values: 1.001 and 5.32 (for Si1), 1.001 and 3.41 (for Si2) as well as 1.005 and 20.08 (for Si3). For polyhedra with perfect holosymmetric shape the values for *λ* and *σ* ^2^ should be 1 and 0, respectively.

**Figure 2:**
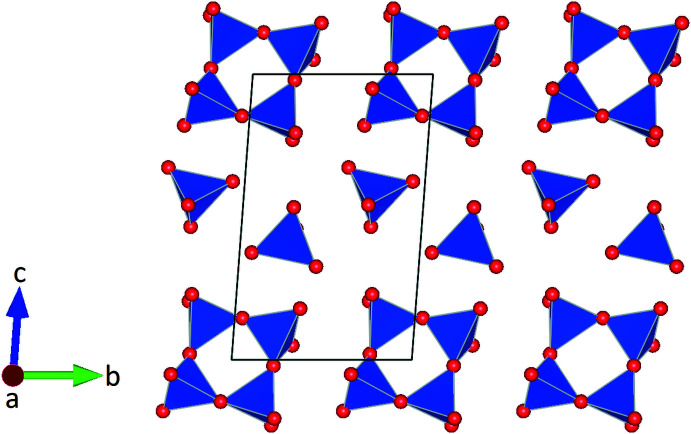
Layer-like arrangement of insular [SiO_4_] tetra­hedra and [Si_4_O_12_] *vierer* single rings in a projection along [100].

**Figure 3:**
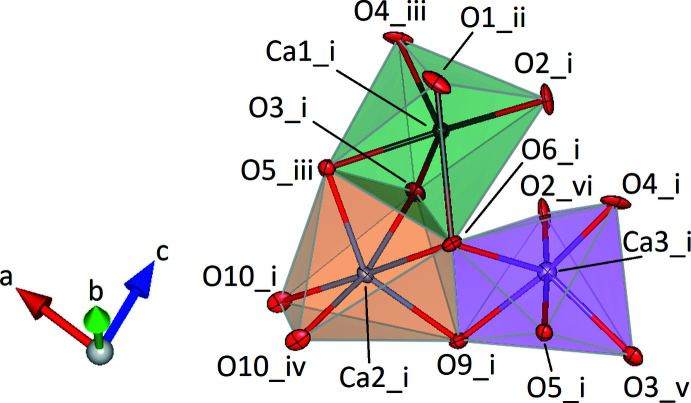
Cluster of three octa­hedra around Ca1 (green), Ca2 (orange) and Ca3 (pink). Oxygen and calcium atoms are shown in red and gray, respectively. Displacement ellipsoids are drawn at the 70% probability level. [Symmetry codes: (i) *x*, *y*, *z*; (ii) *x*, *y* + 1, *z*; (iii) 1 + *x*, *y*, *z*; (iv) 2 − *x*, 1 − *y*, -*z;* (v) −1 + *x*, *y*, *z*; (vi) 1 − *x*,1 − *y*,1 − *z*.]

**Figure 4:**
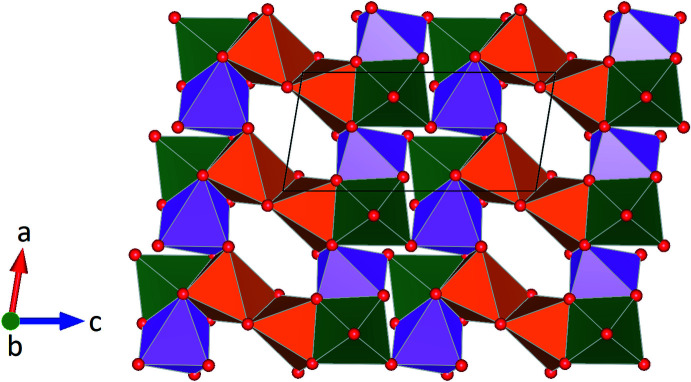
Layer-like structure of [CaO_6_] octa­hedra along [010] (Ca1 in green, Ca2 in orange, Ca3 in pink). Octa­hedra are linked *via* common corners (Ca1–Ca3), edges (Ca2–Ca2 and Ca2–Ca3) and faces (Ca1–Ca2 and Ca1–Ca3)

**Figure 5:**
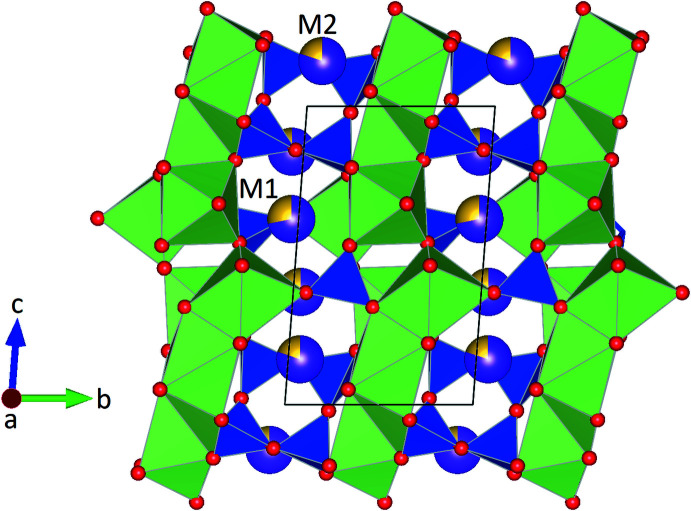
Projection of the crystal structure along [100]. The structure comprises insular as well as cyclic silicate anions (plotted in blue). Corner-, edge- and face-connected [CaO_6_] octa­hedra are painted green. Potassium (violet) and sodium (yellow) cations occupy voids between the [SiO_4_] and [CaO_6_] polyhedra. Bi-colored spheres indicate the mixed K–Na positions (*M*1 and *M*2). The sizes of the bi-colored segments refer to the percentages determined from the site-occupancy refinements.

The silicate anions are located in layers parallel to (100). Within a single layer the *vierer* rings and the isolated tetra­hedra are arranged in rows running parallel to [010] (Fig. 2 ▸). Charge compensation is accomplished by the incorporation of additional Ca^2+^, K^+^ and Na^+^ ions occupying a total of five different crystallographically independent positions. Site population refinements in combination with bond-length considerations indicated that three of them are exclusively occupied with Ca^2+^ cations. They are coordinated by six oxygen ligands in form of distorted octa­hedra (Fig. 3 ▸). The two octa­hedra around Ca1 and Ca3 form a dimer sharing one common face. Each two of these dimers share common corners and are connected into a tetra­mer. By sharing a common edge, two adjacent [Ca2O_6_] octa­hedra build a second type of dimer. These [Ca_2_O_10_] moieties in turn provide linkage between the surrounding tetra­mers by sharing common faces and edges. The complex arrangement of the different [CaO_6_] polyhedra results in the formation of an octa­hedral layer-like building unit extending parallel to (010) (Fig. 4 ▸). The remaining two sites within the asymmetric unit show mixed K/Na populations. They are located between the silicate anions and the [CaO_6_] octa­hedra and have eight and nine next oxygen neighbors. A projection of the crystal structure parallel [100] is given in Fig. 5 ▸. Bond-valence-sum calculations based on the parameter sets of Brese & O’Keeffe (1991 ▸) (for Si—O bonds) and Brown & Altermatt (1985 ▸) (for Ca—O, K—O and Na—O inter­actions) resulted in the following values (in valence units): Si1: 3.884; Si2: 4.062; Si3: 4.143; Ca1: 2.132; Ca2: 1.978; Ca3: 1.858; K1/Na1: 1.073 and K2/Na2: 1.189.

## 3. Database survey

As mentioned above, the title compound is isotypic with K_2_Ca_3_Si_3_O_10_ (Schmidmair *et al.*, 2015 ▸). The main difference is due to the mixed K–Na occupancies of the two inter­stitial cation positions, which are exclusively occupied by K in the corresponding potassium calcium silicate. For the calculation of several qu­anti­tative descriptors for the characterization of the degree of similarity, the program *COMPSTRU* (de la Flor *et al.*, 2016 ▸) was employed. The structure of (K_1.5_Na_0.5_)Ca_3_Si_3_O_10_ was transformed to the most similar configuration of K_2_Ca_3_Si_3_O_10_. The calculations revealed the following displacements (in Å) between the corresponding atom pairs in both phases: Si1: 0.039; Si2: 0.100; Si3: 0.039; Ca1: 0.050; Ca2: 0.107; Ca3: 0.107; (K/Na)1: 0.043; (K/Na)2: 0.133; O1: 0.052; O2: 0.118; O3: 0.046; O4: 0.140; O5: 0.078; O6: 0.084; O7: 0.213; O8: 0.214; O9: 0.181; O10: 0.036. The measure of similarity (Δ) as defined by Bergerhoff *et al.* (1999 ▸) has a value of 0.038. Notably, the most pronounced shifts occur between the two symmetrically independent bridging oxygen atoms of the *vierer* ring (O7, O8).

## 4. Compositional strain

**Figure 6:**
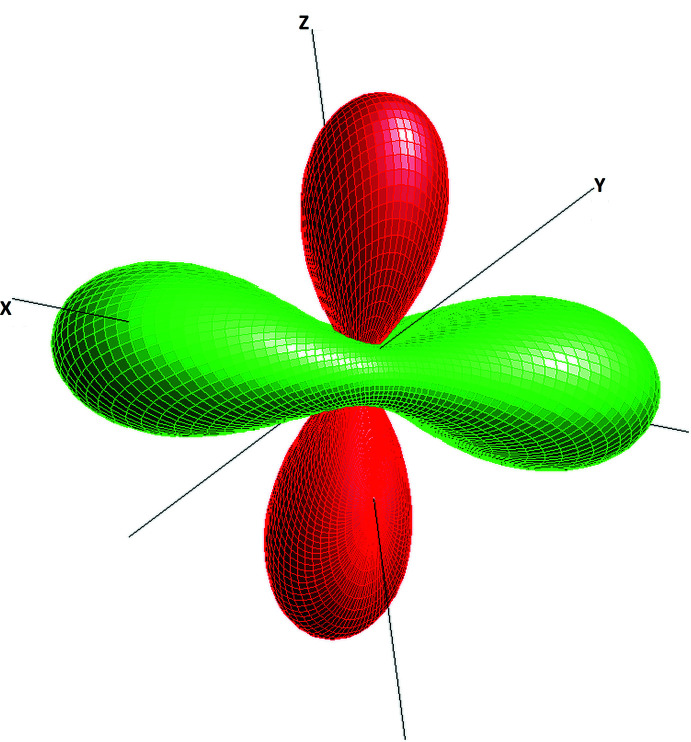
Representation surface of the compositional strain tensor with respect to the underlying Cartesian coordinate system. Green and red parts of the surface indicate positive and negative values, respectively.

The comparison of the unit-cell volumes of (K_1.5_Na_0.5_)Ca_3_Si_3_O_10_ and K_2_Ca_3_Si_3_O_10_ reveals a reduction of about 2.41 Å^3^ or 0.5% for the present compound. On the one hand, this trend has to be expected when larger K^+^ cations are partially replaced with smaller Na^+^ cations. On the other hand, the observed difference in unit-cell volume is not that pronounced. A closer look at the individual lattice parameters reveals two opposing trends. While the *a*-axis direction in the mixed crystal decreases by about 0.04 Å, the *c*-axis increases by 0.05 Å. The *b*-axis direction is virtually unaffected by the K–Na substitution (reduction of 0.003 Å). Furthermore, all three unit-cell angles narrow. Not surprisingly, for low-symmetry solid solutions a complex inter­play between chemical composition and structural changes related to individual deformations of the coordination polyhedra is to be expected. To obtain a more holistic picture of the distortion patterns, the evaluation of the the so-called compositional strain tensor can be a helpful tool. The derivation of the components of this second-rank tensor from two sets of lattice parameters corresponding to two different compositions has been described by Ohashi & Burnham (1973 ▸), for example. The necessary calculations have been performed with the program *Win_Strain* 4.11 (Angel, 2011 ▸). Using a finite Eulerian strain formalism referred to an orthonormal coordinate system {**x**, **y** and **z**} with **z** // **c**, **x** // a* and **y** = **z** × **x**, the following components of the 3 × 3 matrix for the strain tensor ɛ_ij_ were derived: ɛ_11_ = 0.0083 (2); ɛ_22_ = 0.0012 (2); ɛ_33_ = −0.0045 (2); ɛ_12_ = −0.0026 (2); ɛ_13_ = −0.0006 (2) and ɛ_23_ = −0.0058 (2). With respect to the Cartesian coordinate system of the principal axes {**e_1_**, **e_2_** and **e_3_**}, the following three principal strains are obtained: ɛ_1_ = −0.0083 (2); ɛ_2_ = 0.0041 (3); ɛ_3_ = 0.0093 (2) indicating a pronounced anisotropy of the strain. With the help of the symmetrical ɛ_ij_ tensor, the relevant strain can be calculated for any direction defined by a vector **q** whose three components are the direction cosines q_1_, q_2_ and q_3_, *i.e*. the cosines of the angles between **q** and the three axes of the Cartesian reference system. By plotting the individual values as a function of **q**, one obtains a geometric representation of the tensor in form of a surface in three-dimensional space. The visualization of the corresponding surface is given in Fig. 6 ▸. It gives concise information about the distribution of expanding and shrinking directions when the mixed-crystal as the reference state is compared with the pure potassium end-member.

As expected for the triclinic case, the principal axes are not related to the directions of the crystallographic coordinate system along **a**, **b** and **c**. Using *Win_Strain*, the following angles between the principal and the crystallographic axes have been derived. The values given in parentheses refer to the corresponding angles with **a**, **b** and **c**, respectively: **e_1_**: (75.1°; 53.7°; 33.3°); **e_2_**: (72.5°; 44.4°; 122.5°); **e_3_**: (156.7°; 68.0°; 96.9°). Finally, the components of the principal axes in the crystallographic coordinate system have been calculated and the corresponding vectors analyzed together with specific elements of the structure using the program *VESTA-3* (Momma & Izumi, 2011 ▸). Notably, the direction ɛ_3_ of major expansion with increasing K concentration (major compression with increasing Na content) is roughly perpendicular to the mean plane of the *vierer* single rings. The direction of the negative principal axis ɛ_1_ is parallel to the direction of the face-sharing octa­hedral dimers around Ca1 and Ca2.

## 5. Synthesis and initial characterization

Synthesis experiments for a sample with nominal composition (K_1.5_Na_0.5_)Ca_3_Si_3_O_10_ were based on the following fine chemicals: Na_2_CO_3_ (Merck, 99.9%), CaCO_3_ (Merck, >99.9%), K_2_CO_3_ (Alfa Aesar, 99.997%) and SiO_2_ (Alfa Aesar, 99.995%). Before weighing on an analytical balance (Mettler Toledo LabStyle 204), the educts were dried at 573 K for 12 h. A total of 1 g of the stoichiometric mixture was homogenized for 45 min in a planetary ball mill (Fritsch Pulverisette 7) under ethanol. Subsequently, the sample was dried again at 333 K for complete removal of the alcohol and, finally, stored in a desiccator. High-temperature treatment was performed in a small platinum capsule having an inner diameter of 5 mm and a length of 35 mm. After closing the lower end of the capsule with a welding apparatus, about 100 mg of the educt mixture was charged into the Pt container. The capsule was placed vertically in an alumina combustion boat, transferred to a chamber furnace, heated slowly to 973 K and annealed for 12 h for complete disintegration of the carbonates. After removing from the furnace, the solid material was carefully compacted. Subsequently, the upper open end of the capsule was pinched and welded shut. Actually, sealing was performed in order to prevent potassium and/or sodium losses which are likely to occur at temperatures above 1273 K. Subsequently, the container was placed back into the furnace and heated from 298 K to 1573 K with a ramp of 5 K min^−1^. After holding the target temperature for 24 h, the sample was quenched in water. The resulting glass was finally annealed for 24 h at 1273 K and cooled in air. Weighing the closed capsule before and after the high-temperature treatment indicated that the container had not leaked during the synthesis run. The capsule was opened, the solidified melt cake was mechanically separated from the container, further crushed in an agate mortar and transferred to a glass slide under a polarizing binocular. A first inspection revealed the presence of transparent, colorless, birefringent single crystals up to 150 µm in size, showing sharp extinction between crossed polarizers. Several crystalline fragments were fixed on glass fibers using nail hardener. Diffraction experiments aiming on the determination of the unit-cell parameters proved the presence of a compound related to K_2_Ca_3_Si_3_O_10_. The crystal with the best overall diffraction quality was finally selected for structural investigations.

## 6. Refinement

Crystal data, data collection and structure refinement details are summarized in Table 1 ▸. Initial coordinates for the refinement calculations were taken from the isostructural ambient pressure polymorph of K_2_Ca_3_Si_3_O_10_ (Schmidmair *et al.*, 2015 ▸). Unconstrained site-population refinements of the relevant K/Na sites under the assumption of full occupancy resulted in an almost ideal K:Na ratio of 3:1. Therefore, for the final refinement cycles a restraint (SUMP instruction in *SHELXL97*; Sheldrick, 2008 ▸) was introduced which fixed the total K:Na content to 3:1 atoms in the unit cell.

## Supplementary Material

Crystal structure: contains datablock(s) global, I. DOI: 10.1107/S2056989022006193/wm5651sup1.cif

Structure factors: contains datablock(s) I. DOI: 10.1107/S2056989022006193/wm5651Isup2.hkl

CCDC reference: 2178743

Additional supporting information:  crystallographic information; 3D view; checkCIF report

## Figures and Tables

**Table 1:** Experimental details

Crystal data	
Chemical formula	(K_1.5_Na_0.5_)Ca_3_Si_3_O_10_
*M*_r_	435.0
Crystal system, space group	Triclinic, *P* 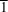
Temperature (K)	296
*a*, *b*, *c* (Å)	5.6269 (12), 7.3728 (16), 11.884 (2)
α, β, γ (°)	85.512 (16), 80.428 (16), 88.275 (18)
*V* (Å^3^)	484.59 (17)
*Z*	2
Radiation type	Mo *K*α
μ (mm^−1^)	2.80
Crystal size (mm)	0.13 × 0.11 × 0.08

Data collection	
Diffractometer	Rigaku Oxford Diffraction Gemini-R Ultra
Absorption correction	Multi-scan (*CrysAlis PRO*; Rigaku OD, 2020 ▸)
*T*_min_, *T*_max_	0.983, 1
No. of measured, independent and observed [*I* > 2σ(*I*)] reflections	3075, 1803, 1136
*R*_int_	0.061
(sin θ/λ)_max_ (Å^−1^)	0.611

Refinement	
*R*[*F* ^2^ > 2σ(*F* ^2^)], *wR*(*F* ^2^), *S*	0.048, 0.095, 0.93
No. of reflections	1803
No. of parameters	166
Δρ_max_, Δρ_min_ (e Å^−3^)	0.65, −0.70

Computer programs: *CrysAlis PRO* (Rigaku OD, 2020 ▸), *SHELXL97* (Sheldrick, 2008 ▸), *VESTA-3* (Momma & Izumi, 2011 ▸), *publCIF* (Westrip, 2010 ▸) and *WinGX* (Farrugia, 2012 ▸).

## supplementary crystallographic information

### Crystal data

**Table:**

(K_1.5_Na_0.5_)Ca_3_Si_3_O_10_	*Z* = 2
*M**_r_* = 435.0	*F*(000) = 432
Triclinic, *P*1	*D*_x_ = 2.981 Mg m^−^^3^
Hall symbol: -P 1	Mo *K*α radiation, λ = 0.71073 Å
*a* = 5.6269 (12) Å	Cell parameters from 395 reflections
*b* = 7.3728 (16) Å	θ = 7.5–26.7°
*c* = 11.884 (2) Å	µ = 2.80 mm^−^^1^
α = 85.512 (16)°	*T* = 296 K
β = 80.428 (16)°	Fragment, colourless
γ = 88.275 (18)°	0.13 × 0.11 × 0.08 mm
*V* = 484.59 (17) Å^3^	

### Data collection

**Table:**

Rigaku Oxford Diffraction Gemini-R Ultra diffractometer	1803 independent reflections
Radiation source: fine-focus sealed X-ray tube, Enhance (Mo) X-ray Source	1136 reflections with *I* > 2σ(*I*)
Graphite monochromator	*R*_int_ = 0.061
Detector resolution: 10.3575 pixels mm^-1^	θ_max_ = 25.8°, θ_min_ = 3.4°
ω (1° width) scans	*h* = −5→6
Absorption correction: multi-scan (*CrysAlisPro*; Rigaku OD, 2020)	*k* = −8→8
*T*_min_ = 0.983, *T*_max_ = 1	*l* = −12→14
3075 measured reflections	

### Refinement

**Table:**

Refinement on *F*^2^	Primary atom site location: structure-invariant direct methods
Least-squares matrix: full	Secondary atom site location: difference Fourier map
*R*[*F*^2^ > 2σ(*F*^2^)] = 0.048	*w* = 1/[σ^2^(*F*_o_^2^) + (0.*P*)^2^] where *P* = (*F*_o_^2^ + 2*F*_c_^2^)/3
*wR*(*F*^2^) = 0.095	(Δ/σ)_max_ < 0.001
*S* = 0.93	Δρ_max_ = 0.65 e Å^−^^3^
1803 reflections	Δρ_min_ = −0.70 e Å^−^^3^
166 parameters	Extinction correction: SHELXL, Fc^*^=kFc[1+0.001xFc^2^λ^3^/sin(2θ)]^-1/4^
0 restraints	Extinction coefficient: 0.0051 (9)

### Special details

**Table:**

Geometry. All s.u.'s (except the s.u. in the dihedral angle between two l.s. planes) are estimated using the full covariance matrix. The cell s.u.'s are taken into account individually in the estimation of s.u.'s in distances, angles and torsion angles; correlations between s.u.'s in cell parameters are only used when they are defined by crystal symmetry. An approximate (isotropic) treatment of cell s.u.'s is used for estimating s.u.'s involving l.s. planes.
Refinement. Refinement of F^2^ against ALL reflections. The weighted R-factor wR and goodness of fit S are based on F^2^, conventional R-factors R are based on F, with F set to zero for negative F^2^. The threshold expression of F^2^ > 2σ(F^2^) is used only for calculating R-factors(gt) etc. and is not relevant to the choice of reflections for refinement. R-factors based on F^2^ are statistically about twice as large as those based on F, and R- factors based on ALL data will be even larger.

### Fractional atomic coordinates and isotropic or equivalent isotropic displacement parameters (Å^2^)

**Table:**

	*x*	*y*	*z*	*U*_iso_*/*U*_eq_	Occ. (<1)
Si1	0.7505 (4)	0.2700 (2)	0.42127 (14)	0.0064 (4)	
Si2	0.3436 (3)	0.8195 (2)	0.15169 (14)	0.0057 (4)	
Si3	0.6353 (4)	0.7469 (2)	−0.09005 (14)	0.0063 (4)	
Ca1	0.7935 (3)	0.75603 (18)	0.37520 (10)	0.0078 (4)	
Ca2	0.8349 (3)	0.53250 (18)	0.13875 (10)	0.0077 (4)	
Ca3	0.2749 (3)	0.50391 (18)	0.33919 (10)	0.0088 (4)	
K1	0.2761 (3)	0.0390 (2)	0.37787 (13)	0.0147 (8)	0.722 (18)
K2	0.8337 (3)	0.0630 (2)	0.15192 (14)	0.0187 (7)	0.799 (17)
Na1	0.2761 (3)	0.0390 (2)	0.37787 (13)	0.0147 (8)	0.278 (18)
Na2	0.8337 (3)	0.0630 (2)	0.15192 (14)	0.0187 (7)	0.201 (17)
O1	0.7932 (10)	0.0677 (6)	0.3755 (3)	0.0160 (12)	
O2	0.5384 (9)	0.6946 (6)	0.5425 (3)	0.0141 (12)	
O3	0.8687 (9)	0.4303 (6)	0.3269 (3)	0.0118 (11)	
O4	0.1173 (9)	0.7175 (7)	0.4662 (3)	0.0137 (12)	
O5	0.0887 (8)	0.7390 (6)	0.2054 (3)	0.0086 (11)	
O6	0.5451 (8)	0.7526 (6)	0.2295 (3)	0.0081 (11)	
O7	0.4227 (9)	0.7652 (7)	0.0206 (3)	0.0183 (13)	
O8	0.6806 (9)	0.9569 (6)	−0.1462 (4)	0.0167 (12)	
O9	0.4718 (9)	0.3596 (6)	0.1794 (3)	0.0112 (11)	
O10	0.8778 (9)	0.6693 (6)	−0.0507 (3)	0.0114 (11)	

### Atomic displacement parameters (Å^2^)

**Table:**

	*U*^11^	*U*^22^	*U*^33^	*U*^12^	*U*^13^	*U*^23^
Si1	0.0067 (11)	0.0044 (9)	0.0081 (9)	0.0023 (8)	−0.0017 (8)	−0.0006 (7)
Si2	0.0051 (11)	0.0060 (9)	0.0056 (8)	0.0006 (8)	−0.0003 (7)	0.0007 (7)
Si3	0.0064 (11)	0.0057 (10)	0.0064 (8)	0.0014 (8)	−0.0009 (7)	0.0007 (7)
Ca1	0.0076 (8)	0.0084 (7)	0.0076 (6)	0.0013 (6)	−0.0019 (6)	−0.0009 (5)
Ca2	0.0092 (8)	0.0084 (7)	0.0054 (6)	0.0005 (6)	−0.0010 (5)	−0.0008 (5)
Ca3	0.0100 (9)	0.0067 (7)	0.0097 (7)	0.0011 (6)	−0.0019 (6)	0.0001 (6)
K1	0.0169 (13)	0.0110 (11)	0.0182 (11)	−0.0014 (8)	−0.0079 (8)	−0.0029 (7)
K2	0.0092 (12)	0.0192 (11)	0.0258 (11)	0.0009 (8)	−0.0023 (8)	0.0084 (8)
Na1	0.0169 (13)	0.0110 (11)	0.0182 (11)	−0.0014 (8)	−0.0079 (8)	−0.0029 (7)
Na2	0.0092 (12)	0.0192 (11)	0.0258 (11)	0.0009 (8)	−0.0023 (8)	0.0084 (8)
O1	0.030 (4)	0.009 (3)	0.009 (2)	0.003 (2)	−0.003 (2)	−0.0027 (19)
O2	0.011 (3)	0.016 (3)	0.012 (2)	0.005 (2)	0.007 (2)	0.002 (2)
O3	0.014 (3)	0.016 (3)	0.006 (2)	−0.001 (2)	−0.002 (2)	0.0024 (19)
O4	0.019 (3)	0.016 (3)	0.010 (2)	0.007 (2)	−0.012 (2)	−0.004 (2)
O5	0.008 (3)	0.011 (3)	0.007 (2)	0.000 (2)	−0.0015 (19)	0.0013 (19)
O6	0.007 (3)	0.008 (2)	0.011 (2)	−0.001 (2)	−0.0037 (19)	−0.0009 (19)
O7	0.010 (3)	0.032 (3)	0.012 (2)	−0.001 (2)	−0.001 (2)	−0.005 (2)
O8	0.014 (3)	0.006 (3)	0.029 (3)	−0.001 (2)	−0.001 (2)	0.001 (2)
O9	0.008 (3)	0.015 (3)	0.012 (2)	−0.001 (2)	−0.003 (2)	−0.005 (2)
O10	0.013 (3)	0.009 (3)	0.013 (2)	0.000 (2)	−0.007 (2)	0.0053 (19)

### Geometric parameters (Å, º)

**Table:**

Si1—O1	1.625 (5)	Ca2—O6	2.447 (5)
Si1—O2^i^	1.630 (5)	Ca3—O2^i^	2.302 (5)
Si1—O3	1.640 (4)	Ca3—O4	2.321 (5)
Si1—O4^i^	1.645 (5)	Ca3—O9	2.347 (4)
Si2—O5	1.583 (5)	Ca3—O3^vii^	2.396 (5)
Si2—O6	1.621 (5)	Ca3—O6	2.551 (4)
Si2—O7	1.625 (5)	Ca3—O5	2.578 (5)
Si2—O8^ii^	1.647 (5)	K1—O2^i^	2.566 (5)
Si3—O9^iii^	1.575 (5)	K1—O4^viii^	2.636 (5)
Si3—O10	1.591 (5)	K1—O8^iii^	2.721 (5)
Si3—O7	1.635 (5)	K1—O1^vii^	2.724 (6)
Si3—O8	1.648 (5)	K1—O1	2.919 (6)
Ca1—O4^iv^	2.268 (5)	K1—O1^ix^	2.940 (4)
Ca1—O2	2.273 (4)	K1—O6^viii^	3.060 (5)
Ca1—O1^v^	2.298 (5)	K2—O1	2.631 (4)
Ca1—O5^iv^	2.400 (4)	K2—O10^vi^	2.674 (5)
Ca1—O6	2.401 (5)	K2—O8^vi^	2.722 (6)
Ca1—O3	2.520 (5)	K2—O5^x^	2.834 (5)
Ca2—O10^vi^	2.336 (5)	K2—O6^viii^	2.850 (5)
Ca2—O3	2.339 (4)	K2—O7^iii^	2.891 (5)
Ca2—O10	2.371 (4)	K2—O8^iii^	2.915 (6)
Ca2—O5^iv^	2.383 (5)	K2—O9	2.942 (5)
Ca2—O9	2.401 (5)		
			
O1—Si1—O2^i^	108.4 (3)	O10^vi^—Ca2—O3	96.18 (17)
O1—Si1—O3	112.9 (2)	O10^vi^—Ca2—O10	81.53 (18)
O2^i^—Si1—O3	110.6 (3)	O3—Ca2—O10	167.70 (18)
O1—Si1—O4^i^	109.5 (3)	O10^vi^—Ca2—O5^iv^	100.65 (17)
O2^i^—Si1—O4^i^	109.3 (2)	O3—Ca2—O5^iv^	72.82 (15)
O3—Si1—O4^i^	106.0 (3)	O10—Ca2—O5^iv^	95.65 (15)
O5—Si2—O6	110.8 (2)	O10^vi^—Ca2—O9	103.91 (17)
O5—Si2—O7	110.8 (3)	O3—Ca2—O9	82.88 (16)
O6—Si2—O7	111.2 (3)	O10—Ca2—O9	109.41 (16)
O5—Si2—O8^ii^	108.0 (3)	O5^iv^—Ca2—O9	147.01 (15)
O6—Si2—O8^ii^	109.2 (3)	O10^vi^—Ca2—O6	177.90 (17)
O7—Si2—O8^ii^	106.6 (3)	O3—Ca2—O6	84.25 (16)
O9^iii^—Si3—O10	117.6 (3)	O10—Ca2—O6	97.62 (16)
O9^iii^—Si3—O7	107.4 (3)	O5^iv^—Ca2—O6	77.50 (16)
O10—Si3—O7	110.6 (3)	O9—Ca2—O6	78.18 (16)
O9^iii^—Si3—O8	107.0 (3)	O2^i^—Ca3—O4	100.44 (16)
O10—Si3—O8	108.6 (3)	O2^i^—Ca3—O9	90.05 (17)
O7—Si3—O8	104.8 (3)	O4—Ca3—O9	164.28 (17)
O4^iv^—Ca1—O2	90.97 (17)	O2^i^—Ca3—O3^vii^	115.01 (16)
O4^iv^—Ca1—O1^v^	93.65 (19)	O4—Ca3—O3^vii^	87.68 (18)
O2—Ca1—O1^v^	98.53 (17)	O9—Ca3—O3^vii^	98.54 (17)
O4^iv^—Ca1—O5^iv^	83.90 (16)	O2^i^—Ca3—O6	114.66 (18)
O2—Ca1—O5^iv^	165.18 (17)	O4—Ca3—O6	87.83 (15)
O1^v^—Ca1—O5^iv^	95.68 (16)	O9—Ca3—O6	77.10 (15)
O4^iv^—Ca1—O6	160.71 (16)	O3^vii^—Ca3—O6	130.11 (16)
O2—Ca1—O6	104.81 (17)	O2^i^—Ca3—O5	176.47 (17)
O1^v^—Ca1—O6	94.87 (17)	O4—Ca3—O5	78.99 (15)
O5^iv^—Ca1—O6	78.06 (15)	O9—Ca3—O5	89.82 (15)
O4^iv^—Ca1—O3	85.90 (18)	O3^vii^—Ca3—O5	68.49 (15)
O2—Ca1—O3	96.41 (16)	O6—Ca3—O5	61.88 (15)
O1^v^—Ca1—O3	165.06 (15)	Si2—O7—Si3	148.7 (4)
O5^iv^—Ca1—O3	69.41 (14)	Si2^ii^—O8—Si3	156.4 (3)
O6—Ca1—O3	81.43 (16)		

Symmetry codes: (i) −*x*+1, −*y*+1, −*z*+1; (ii) −*x*+1, −*y*+2, −*z*; (iii) −*x*+1, −*y*+1, −*z*; (iv) *x*+1, *y*, *z*; (v) *x*, *y*+1, *z*; (vi) −*x*+2, −*y*+1, −*z*; (vii) *x*−1, *y*, *z*; (viii) *x*, *y*−1, *z*; (ix) −*x*+1, −*y*, −*z*+1; (x) *x*+1, *y*−1, *z*.
